# Desmoplasia and oncogene driven acinar-to-ductal metaplasia are concurrent events during acinar cell-derived pancreatic cancer initiation in young adult mice

**DOI:** 10.1371/journal.pone.0221810

**Published:** 2019-09-06

**Authors:** Benjamin L. Johnson, Marcela d’Alincourt Salazar, Sarah Mackenzie-Dyck, Massimo D’Apuzzo, Hung Ping Shih, Edwin R. Manuel, Don J. Diamond

**Affiliations:** 1 Department of Hematology and Hematopoietic Cell Transplantation, Beckman Research Institute of City of Hope, Duarte, CA, United States of America; 2 Irell & Manella Graduate School of Biological Sciences, Beckman Research Institute of City of Hope, Duarte, CA, United States of America; 3 Department of Pathology, City of Hope Comprehensive Cancer Center, Duarte, CA, United States of America; 4 Department of Translational Research and Cellular Therapeutics, Diabetes and Metabolic Research Institute, Beckman Research Institute of City of Hope, Duarte, CA, United States of America; 5 Department of Immuno-Oncology, Beckman Research Institute of City of Hope, Duarte, CA, United States of America; Centro Nacional de Investigaciones Oncologicas, SPAIN

## Abstract

The five-year survival rate of patients diagnosed with advanced pancreatic ductal adenocarcinoma (PDAC) has remained static at <5% despite decades of research. With the exception of erlotinib, clinical trials have failed to demonstrate the benefit of any targeted therapy for PDAC despite promising results in preclinical animal studies. The development of more refined mouse models of PDAC which recapitulate the carcinogenic progression from non-neoplastic, adult exocrine subsets of pancreatic cells to invasive carcinoma in humans are needed to facilitate the accurate translation of therapies to the clinic. To study acinar cell-derived PDAC initiation, we developed a genetically engineered mouse model of PDAC, called KPT, utilizing a tamoxifen-inducible Cre recombinase/estrogen receptor (ESR1) fusion protein knocked into the *Ptf1a* locus to activate the expression of oncogenic *Kras*^*G12D*^ and *Trp53*^*R270H*^ alleles in mature pancreatic acinar cells. Oncogene-expressing acinar cells underwent acinar-to-ductal metaplasia, and formed pancreatic intraepithelial neoplasia lesions following the induction of oncogene expression. After a defined latency period, oncogene-expressing acinar cells initiated the formation of highly differentiated and fibrotic tumors, which metastasized to the lungs and liver. Whole-transcriptome analysis of microdissected regions of acinar-to-ductal metaplasia and histological validation experiments demonstrated that regions of acinar-to-ductal metaplasia are characterized by the deposition of the extracellular matrix component hyaluronan. These results indicate that acinar cells expressing *Kras*^*G12D*^ and *Trp53*^*R270H*^ can initiate PDAC development in young adult mice and implicate hyaluronan deposition in the formation of the earliest characterized PDAC precursor lesions (and the progression of pancreatic cancer). Further studies are necessary to provide a comprehensive characterization of PDAC progression and treatment response in KPT mice and to investigate whether the KPT model could be used as a tool to study translational aspects of acinar cell-derived PDAC tumorigenesis.

## Introduction

Pancreatic cancer is currently the fourth leading cause of cancer-related mortality in the United States and is projected to become the second leading cause of cancer related death in the United States and Europe by 2030 [1]. The majority of pancreatic cancer patients are diagnosed with metastatic pancreatic ductal adenocarcinoma (PDAC) and metastatic pancreatic cancer patients have a dismal 5-year survival rate of 2.7% [2,3]. However, early detection of PDAC dramatically improves the 5-year survival rate to 34.3% for the <10% of patients who are diagnosed with early stage, surgically resectable disease [2,4]. Early disease detection and treatment dramatically extends patient survival; consequently there is an unmet need to expand the utility of research tools and mouse models of PDAC to continue to develop approaches that could enable the identification of early disease biomarkers and therapeutic targets.

Most human PDAC tumors (>90%) express constitutively activated KRAS gene variants, and activating KRAS mutations are hypothesized to be the initiating event driving PDAC development [5–7]. Genetic deletions or mutations that abrogate the DNA binding capacity of TP53 occur in 70% of cases of human PDAC, and TP53 mutations are associated with high-grade pancreatic intraepithelial neoplasia (PanIN) lesions and carcinoma *in situ* [8,9]. Studies examining the capacity of acinar and ductal cells (both cellular subsets expressing constitutively active *Kras*^*G12D*^ and lacking functional *Trp53*) in genetically engineered mice indicate that both populations may initiate PDAC formation [10,11]. In these transgenic mouse models of PDAC initiation, oncogene-expressing acinar cells produced abundant precancerous lesions while ductal cell-initiated PDAC was preceded by the formation of rare, high-grade precancerous lesions. To date, several mouse models of PDAC have been developed; for a detailed description of additional models, please refer to references [12,13].

The fibrotic deposition of extracellular matrix components, known as the desmoplastic reaction, accompanies late stage PanIN lesions in mouse models of PDAC [14,15]. Specifically, the accumulation of the extracellular matrix glycosaminoglycan hyaluronanic acid (HA) in the tumor stroma is a hallmark of human PDAC. Desmoplastic HA deposition reduces intratumoral blood perfusion, promotes tumor growth and increases cancer cell motility in humans and animal models of PDAC [16–20]. Transgenic mouse models of PDAC in which expression of oncogenes occurs during embryogenesis recapitulate the late stage hallmarks of human PDAC including desmoplasia, and metastasis to distant organs. However, embryonic activation of oncogenes results in models which fail to accurately represent the initial stages of PDAC carcinogenesis [21].

In this manuscript we investigate the capacity of mature pancreatic acinar cells to initiate the development of PDAC using a lineage tracing approach in the *Kras*^*LSL-G12D/+;*^ *Trp53*^*LSL-R270H/+*^*; Ptf1a*^*CreERTM/+*^*; Rosa26*^*LSL-mCL/LSL-mCL*^ (KPT) mouse model of pancreatic cancer. We demonstrate that mature pancreatic acinar cells expressing oncogenic *Kras*^*G12D*^ and *Trp53*^*R270H*^ transdifferentiate into duct-like cells through the process of acinar-to-ductal metaplasia (ADM) that these cells initiate the development of increasingly abundant PanIN lesions, and ultimately form desmoplastic and metastatic PDAC. We use laser capture microdissection (LCM) and RNA sequencing (RNA-Seq) to generate a transcriptome profile of regions of ADM to identify activated canonical signaling pathways, and gene transcripts that are differentially expressed in both ADM and fully developed PDAC compared to the healthy pancreas. LCM-mediated transcriptional profiling and histological analysis of regions of ADM revealed the deposition of HA specifically in regions of ADM. The results indicate that the KPT model is an alternative platform which may be used to identify the cellular and molecular mechanisms by which acinar cell-derived PDAC progresses.

## Materials and methods

### Animal strains and husbandry

All experiments involving animals were performed in accordance with federal regulations and with the prior approval of the Institutional Animal Care and Use Committee (IACUC) at the City of Hope (protocol number 08048). Both male and female mice were used in this study. Mice were euthanized by CO_2_ inhalation following an IACUC approved protocol in which humane study endpoint criteria were observed. Study endpoint criteria included obvious distress, failure to ambulate, weight loss > 20%, or tumor burden > 500mm^3^ in volume. All experiments were performed in an Association for the Assessment and Accreditation of Laboratory Animal Care (AAALAC) accredited institution. Animals were housed in standard specific-pathogen-free conditions and allowed food and water *ad libitum*. *Ptf1a*^*tm2(cre/ESR1)Cvw/*^*J* (*Ptf1a*^*CreERTM)*^) mice [22], purchased from Jackson Laboratories were cross-breed with *Kras*^*LSL-G12D/+*^*; Trp53*^*LSL-R270H/+*^ *Rosa26*^*LSL-mCL/LSL-mCL*^ mice, previously described and provided by Dr. Thomas Ludwig [23], to generate *Ptf1a*^*CreERTM/+*^; *Kras*^*LSL-G12D/+*^*; Trp53*^*LSL-R270H/+*^*; Rosa26*^*LSL-mCL/LSL-mCL*^ (KPT) and *Ptf1a*^*+/+*^; *Kras*^*LSL-G12D/+*^*; Trp53*^*LSL-R270H/+*^*; Rosa26*^*LSL-mCL/LSL-mCL*^ (KP) mice. KPT/KP mice were maintained on a B6 (Cg)-*Tyr*^*c-2J*^/J, (B6-albino) background. The original development of the *Kras*^*LSL-G12D*^ *and Trp53*^*R270H*^ transgenes used in these studies are detailed in separate publications [24,25].

Tamoxifen (Sigma-Aldrich, Cat# T5648) was dissolved in corn oil, and 4–5-week-old KPT and KP mice of both sexes were administered intraperitoneal injections of 2 mg tamoxifen/ 20g body weight every other day for 5 days.

### Bioluminescence imaging

Mice were intraperitoneally injected with 300 μg D-luciferin potassium salt (Gold Bio, Cat# LUCK-100) dissolved in water 5 minutes prior to imaging. Mice were anesthetized by inhalation of isoflurane and provided supplemental oxygen during imaging. Bioluminescence images were recorded using the LagoX optical imaging systems (Spectral Instruments), set to acquire images with a 10 second exposure time and an open emissions filter.

### Histology, immunofluorescence and histochemistry

Following necropsy, mouse pancreata and pancreatic tumors were fixed by immersion in 10% neutral-buffered formalin solution. Pancreata were then embedded in paraffin and cut into 4 μM sections. Sections were stained with hematoxylin and eosin (H&E) and Masson’s Trichrome by personnel in the Pathology Core Laboratory at City of Hope.

For immunofluorescence staining, tissue sections were deparaffinized by immersion in two changes of xylene. Sections were then rehydrated to 70% ethanol using graded ethanol solutions before incubation in MaxBlock Autofluorescence Reducing Reagent A (MaxVision Biosciences, Cat# MB-M) for 5 minutes. Slides were rinsed in 60% ethanol for 1 minute then distilled water for 5 minutes. Antigen retrieval was performed by immersing slides in 10mM sodium citrate with 0.05% Tween 20, pH 6.0 and heating the buffer solution to 95°C for 20 minutes. Slides were then allowed to cool to room temperature for an hour. Slides were washed in ddH_2_O for 5 minutes, then non-specific antibody binding was blocked by incubating sections with 5% neutral donkey serum (Jackson ImmunoResearch, Cat# 017-000-001) diluted in phosphate buffered saline + 0.05% Tween 20 (PBS-T) for 1 hour at room temperature. Slides were washed 3 times for 5 minutes each with PBS-T, and then incubated with diluted primary antibody overnight at 4°C. Slides were washed with 3 changes of PBS-T, incubated with diluted secondary antibody for 1 hour at room temperature, and again washed with 3 changes of PBS-T. Slides were then washed with distilled water for 5 minutes, incubated with MaxBlock^™^ Autofluorescence Reducing Reagent B (MaxVision Biosciences, Cat# MB-M) for 5 minutes, and then washed 3 times in distilled water for 5 minutes per wash. Nuclei were counterstained with DAPI (4',6-Diamidino-2-Phenylindole, Dihydrochloride), and slides were coversliped using ProLong Diamond Antifade Mountant (Thermo Fisher, Cat# P36965). Primary antibodies were diluted in PBS-T and used at the following concentration CK19 1/20 (DSHB Cat# TROMA-III, RRID:AB_2133570), Cpa1 1/1000 (R&D Systems Cat# AF2765, RRID:AB_2085841), Sox9 1/5000 (Millipore Cat# AB5535, RRID:AB_2239761), αSMA 1/200 (Thermo Fisher Scientific Cat# 14-9760-80, RRID:AB_2572995), mCherry 1/50 (Rockland Cat# 600-401-379, RRID:AB_2209751). The following secondary antibodies were all used at 1/200 dilution to generate fluorescent signal Rhodamine Red-X (RRX) Donkey Anti-Rat IgG (Jackson ImmunoResearch Cat# 712-295-153, RRID:AB_2340676), Alexa Fluor 647 Donkey Anti-Goat (Jackson ImmunoResearch Cat# 705-605-147, RRID:AB_2340437), Alexa Fluor 488 F(ab')_2_ Fragment Donkey Anti-Mouse IgG (Jackson ImmunoResearch Cat# 715-546-151, RRID:AB_2340850), Alexa Fluor 488 Donkey Anti-Goat IgG (Jackson ImmunoResearch Cat# 705-545-147, RRID:AB_2336933), Alexa Fluor 647 Donkey Anti-Rabbit IgG (Jackson ImmunoResearch Cat# 711-605-152, RRID:AB_2492288). The Alexa Fluor 647 Tyramide SuperBoost kit (Invitrogen, Cat# B40926) was used according to manufacturer’s specifications to generate the fluorescent signal for mCherry IF staining. When using mouse derived primary antibodies, slides were incubated with AffiniPure Fab Fragment Donkey Anti-Mouse IgG (Jackson ImmunoResearch Cat# 715-007-003, RRID:AB_2307338) at a final concentration of 20 μg/mL for 1 hour following neutral serum blocking, to block endogenous mouse IgG. Slides were washed with 3 changes of PBS-T for 5 minutes each, following endogenous mouse IgG blocking prior to primary antibody incubation. The TROMA-III hybridoma, developed by Kemler, R. was obtained from the Developmental Studies Hybridoma Bank, created by the NICHD of the NIH and maintained at The University of Iowa, Department of Biology, Iowa City, IA 52242.

### Histochemical visualization of hyaluronan

For histochemical visualization of hyaluronan using biotinylated hyaluronic acid binding protein (HABP) (Millipore, Cat# 385911), tissue sections were deparaffinized in 2 changes of xylene then endogenous peroxidase activity was quenched by incubating tissue in 0.7% H_2_O_2_ in absolute methanol for 20 minutes. Slides were rehydrated in graded ethanol and then incubated with 1% bovine serum albumin (BSA) diluted in PBS-T for 30 minutes to block non-specific protein binding. Blocking solution was decanted and slides were incubated with 5 μg/mL HABP diluted in PBS-T overnight at 4°C. Slides were washed with 3 changes of PBS-T for 5 minutes each, then incubated with Streptavidin HRP (BD Bioscience, Cat# 554066) diluted 1/1000 in PBS-T for one hour. Slides were washed with 3 changes of PBS-T, and then ImmPACT DAB HRP substrate (Vector Labs, Cat# SK-4105) was used to visualize HA staining according to the manufacture’s protocol. Hematoxylin QS (Vector Labs, Cat# H-3404) was used to counterstain nuclei according to the manufacture’s protocol. Slides were then dehydrated through graded ethanol solutions, cleared in two changes of xylene and coverslipped using with VectaMount Permanent Mounting Medium (Vector Labs, Cat# H-5000).

### Microscopy

All fluorescent micrographs were captured using a Leica DMi8 inverted microscope. Fluorescent images were captured using a monochrome Leica DFC365FX digital camera and LasX software (Leica) was used to operate the microscope and capture images. RGB color images were captured using the Zeiss Observer microscope with Zeiss AxioCam 506 Color digital camera, and using the ZEN Blue 2.3 software (Zeiss) to operate the microscope and capture images. Raw images were processed and given scale bars using Image Pro Premier 9.3 software (Media Cybernetics), and resized and formatted in Microsoft PowerPoint. All images presented for comparison were captured using identical microscope acquisition setting. Immunofluorescent images presented for comparison were adjusted to have identical LUT settings, and LUT settings were chosen to reflect the true range of signal present in all samples. The brightness and contrast of images were increased using PowerPoint software, and was performed uniformly on all images presented for comparison.

### Alcian blue staining

Tissue sections were deparaffinized in 2 changes of xylene, and rehydrated through graded ethanol solutions. Alcian blue and eosin (AB/E) stained sections were then incubated for 30 minutes in Alcian blue solution, 1% Alcian blue 8GX (Alfa Aesar, Cat# J6012209) dissolved in a 3% solution of acetic acid. Slides were rinsed in distilled water, dehydrated to 95% ethanol, stained with eosin Y (Thermo Fisher, Cat# 6766007) for 10 seconds, dehydrated through graded ethanol solutions, incubated in anhydrous ethanol, and cleared with 2 changes of xylene and coverslipped using VectaMount Permanent Mounting Medium (Vector Labs, Cat# H-5000). The Zeiss Observer microscope and ZEN Blue 2.3 software were used to generate tile scan images covering the entirety of pancreatic sections. The segmentation module of Image Pro Premier 9.3 software was used to quantify total pancreatic area, and Alcian blue positive area from whole slides.

### Histological classification of pancreatic lesions

Pancreatic tissue was classified histologically, by HE analysis, supplemented by AB/E and immunohistochemistry-stained sections, under the supervision of clinical surgical pathologist (MD’A), as unremarkable or bearing pancreatic acinar to ductal metasplasia (ADM), pancreatic intraepithelial neoplasia (PanIN) or invasive carcinoma. The following minimal criteria where utilized: **1.** ADM was determined by the presence of duct-like cells with abundant mucinous cytoplasm within pancreatic acini and lobules; **2.** PanIN was determined by the presence of flat or architecturally complex intraductal epithelial lesions composed of tall columnar cells with basally located nuclei and abundant supranuclear mucin. Assessment and quantitation of PanIN lesions was aided by analysis of AB/E stained sections; **3.** Invasive carcinoma was diagnosed by the presence of infiltrating, well- to poorly-formed glands displaying marked cytological atypia and high mitotic activity. Invasion was typically associated with a marked desmoplastic response.

### Laser capture microdissection

Three KPT (male: *n =* 1, female: *n =* 2) and KP (male: *n =* 2, female: *n =* 1) mice were euthanized 2 months post-tamoxifen treatment. Resected KP pancreata were washed in ice chilled PBS, then submerged in RNALater (Qiagen, Cat# 76104) and stored at 4°C until RNA extraction. Unfixed KPT pancreata were snap frozen and sectioned onto polyethylene naphthalate (PEN) membrane slides for laser capture microdissection (LCM). Additionally, portions of tumors were isolated from 3 KPT mice (male: *n =* 1; female: *n =* 2) once they had reached humane study endpoint criteria, or approximately 6 months post tamoxifen treatment. KPT tumor fragments were also snap frozen and sectioned onto PEN membrane slides in preparation for LCM. Tissue sections mounted on PEN membrane slides were stained with hematoxylin, and then regions of ADM were identified by their distinctive histological characteristics (metaplastic ductal epithelium) and were isolated via LCM. Additionally in KPT tumor fragments, ductal tumor epithelium was separated from surrounding stromal tissue using LCM. RNA was extracted from both LCM-enriched populations and from the pancreas of KP mice. This resulted in 3 RNA sample groups, each composed of 3 biological replicates: “HP,” total pancreatic RNA isolated from age-matched KP mice; “ADM,” a population enriched for acinar cells undergoing ADM and cells infiltrating regions of ADM; and “PDAC,” enriched for pancreatic tumor cells.

Resected KPT mouse pancreata and KPT tumor fragments were briefly washed in ice chilled PBS, equilibrated to O.C.T. Compound (VWR, Cat# 25608–930) for 2 minutes, and then frozen using liquid nitrogen cooled isopentane. 10 μM sections of tissue were cut using a Cryostat-Microtome, and sections were adhered to PEN membrane slides (Leica, Cat# 11600289) and stored at -80°C. Slides were fixed by immersion in -20°C chilled 70% ethanol for 30 seconds, then transferred to RNase H_2_0 and incubated for 30 seconds. Slides were removed from RNase H_2_0 and excess water was blotted using a Kimwipe, and nuclei were stained with Hematoxylin QS (Vector Labs, Cat# H-3404) for 5 seconds. Slides were incubated in RNAse Free water for 30 seconds, to remove excess staining solution. Slides were dehydrated by incubating in graded ethanol solutions, (1x 70% ethanol, 1x 95% ethanol and 2x 100% ethanol) for 1 minute each, and air dried for 2 minutes. ProtectRNA RNase inhibitor (Sigma, Cat# R7397) was included at 1x concentration in all staining solutions, excluding 100% ethanol. All staining solutions were stored on ice until immediately prior to use, excluding the final 100% ethanol solution which was stored at room temperature. Stained slides were immediately subjected to microdissection using a Leica LMD 7000 microscope equipped with a 349nm solid state LASER and CC7000 digital camera.

### RNA isolation

RNA was extracted from microdissected specimens and KP pancreata using the PicoPure RNA Isolation Kit (Thermo Fisher, Cat# KIT0204) following the manufacturer’s instructions, including the optional on column DNAse digestion using RNase Free DNase (Qiagen, Cat#79254). RNA was eluted using 11 μL of RNase Free ddH_2_O and 0.5 μL of SUPERase•In RNase Inhibitor (Thermo Fisher, Cat# AM2694) was immediately added to eluted RNA. A 1 μL aliquot of each RNA sample was then stored into a separate 0.2 μL Axygen PCR tube (Thermo Fisher, Cat# 14-222-261) and this sample was used for RNA QC. RNA quality was quantified using the Agilent RNA 6000 Pico Chip (Agilent, Cat# 5067–1513) and Agilent 2100 Bioanalyzer platform. RNA integrity number (RIN) for each sample is shown in S1 Table.

### RNA-Seq library preparation and sequencing with Illumina Hiseq2500

RNA-Seq libraries were prepared using the Kapa RNA HyperPrep Kit with RiboErase (Kapa Biosystems, Cat# KR1351) according to the manufacturer's protocol. Twenty five ng of total RNA from each sample was subjected to ribosomal RNA depletion. The hybridization mix was purified and treated with DNase to remove the hybridization oligonucleotides. Purified RNA underwent fragmentation, first-strand cDNA synthesis, and second-strand cDNA synthesis, followed by 3′ end adenylation. Barcoded adaptors were ligated to the adenylated, double-stranded cDNA fragments. 13 cycles of PCR were performed to produce the final sequencing library. cDNA library quality was determined using a High Sensitivity DNA Chip (Agilent, Cat# 5067–4626) with the Agilent 2100 Bioanalyzer platform, and cDNA libraries were quantified using a Qubit fluorometer (Thermo Fisher Scientific). Library templates were prepared for sequencing using the HiSeq SR Cluster v4 Kit (Illumina, Cat# GD-401-4001). Sequencing runs were performed using the Illumina HiSeq 2500 platform with HiSeq SBS v4 Kit (Illumina, Cat# FC-401-4002). The HiSeq Control (HCS 2.2.38) and Real-Time Analysis (RTA 1.18.61) software were used for image analysis and base calling.

### RNA-Seq data analysis

RNA was sequenced using Illumina protocols to generate 50-bp reads. Sequenced reads were aligned to the mouse mm10 reference genome using TopHat2 [26]. Transcript expression levels were quantified by HTSeq [27], and DESeq2 was utilized to identify differentially expressed genes [28]. Transcript abundance was quantified as reads per kilobase of transcript per million fragments mapped (RPKM). RNA-Seq quality control analyzes are shown in **S1 Table**. Transcriptomic signatures of ADM and PDAC were compared to whole normal pancreas. Kras and Trp53 mutational analyzes confirming that the RNA-Seq studies were performed on true oncogenic lesions are shown in **S2 Table**. Validation of RNA-Seq data by real-time quantitative PCR (qPCR) and primer list used are described in **S2 Fig**. The Benjamini-Hochberg procedure was used to adjust *P* values for false discovery rate. Pathway analysis was performed with Ingenuity Pathway Analysis (IPA, Qiagen).

Complete RNA-Seq data including raw and processed data files is available online through the Gene Expression Omnibus (https://www.ncbi.nlm.nih.gov/geo/) GenBank Accession Number GSE111540.

### Statistical analysis

Statistical significance in survival analysis was determined using the log-rank test. Statistical analysis of Alcian blue-positive regions was performed using two-tailed unpaired student’s *t* tests. All statistical analyses were performed using Graph Pad Prism software v. 7.03.

## Results

### *Ptf1a*^*CreERTM*^-mediated recombination is restricted to mature pancreatic acinar cells

**Fig 1:**
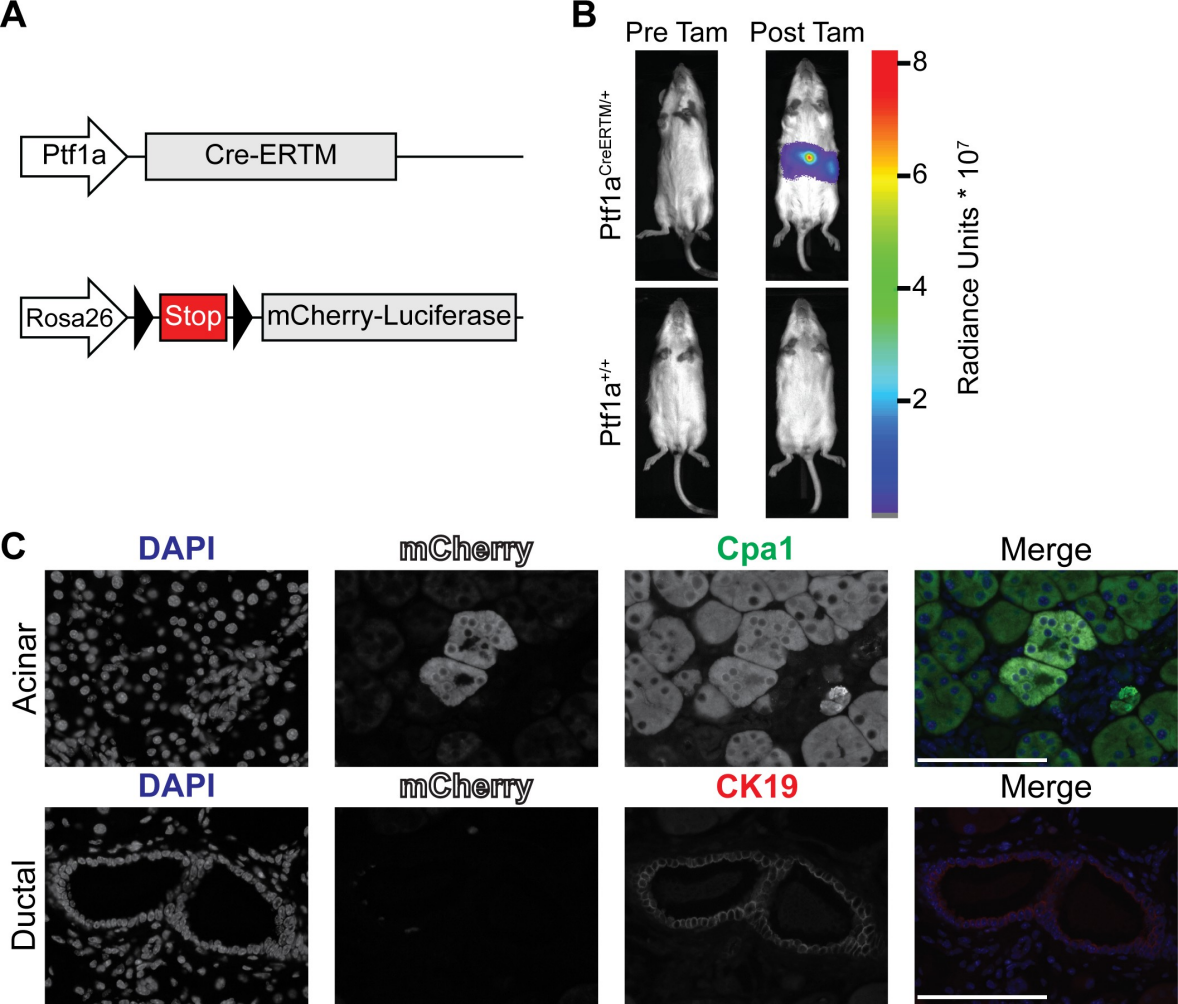
Ptf1aCreERTM mediated recombination is pancreatic acinar cell specific and occurs only following tamoxifen administration. **(A)** Graphic representation of alleles altered in Ptf1aCreERTM mice. Triangles represent LoxP sites. Arrows indicate genetic loci. Grey boxes indicate exons. **(B)** Bioluminescence imaging demonstrating tamoxifen (Tam) induced Ptf1aCreERTM activity; images are representative of 3 separate experiments involving 2 mice per group. Radiance unit is photons/second/cm2/steradian **(C)** Multiplexed IF micrographs indicating expression of lineage marker (mCherry) only in acinar cells. Micrographs depict acinar cells (upper panels) and pancreatic ductal cells (lower panels). Channels are DAPI (blue), mCherry (white), Cpa1 (green), and CK19 (red). Micrographs are representative of *n* = 2 Ptf1aCre-ERTM positive mice. Magnification 63X, scale bars represent 100 μm.

To generate an acinar cell specific model of PDAC, we evaluated the capacity of Cre recombinase to induce the expression of oncogenes specifically in mature pancreatic acinar cells. We determined if a tamoxifen-inducible Cre recombinase estrogen receptor fusion protein (CreERTM) inserted into the *Ptf1a* locus (*Ptf1a*^*CreERTM*^) restricts Cre-mediated recombination to mature pancreatic acinar cells. The *Rosa26*^*LSL-mCL*^ transgene was used to irreversibly label cells in which Cre-mediated recombination had occurred with an mCherry-luciferase fusion protein (mCL) (**Fig 1A**). The ability of the *Ptf1a*^*CreERTM*^ transgene to activate expression of the mCL reporter construct *in vivo* was determined by injecting 4–5-week-old mice with tamoxifen, and evaluating luciferase expression using bioluminescence imaging. **Fig 1B** depicts representative bioluminescence images of *Ptf1a*^*CreERTM/+*^*; Rosa26*^*LSL-mCL/LSL-mCL*^ and *Ptf1a*^*+/+*^; *Rosa26*^*LSL-mCL/LSL-mCL*^ mice immediately prior to and 1 week post-tamoxifen injections (*n* = 2 mice per genotype, 3 separate experiments). No luciferase signal was observed in control *Ptf1a*^*+/+*^ mice before or after tamoxifen treatment. Furthermore, *Ptf1a*^*CreERTM*^ activity, indicated by luciferase signal, was not observed in any mice prior to tamoxifen treatment. Multiplexed immunofluorescence (IF) staining using antibodies against mCherry was used to determine the pancreatic cell type in which CreERTM-mediated recombination had occurred (**Fig 1C**). Carboxypeptidase A1 (Cpa1) and Keratin 19 (CK19) were used as molecular markers for pancreatic acinar and ductal cells, respectively. mCherry and Cpa1 double positive acinar cells were observed in the pancreata of *Ptf1a*^*CreERTM/+*^ animals, whereas mCherry and CK19 double-positive cells were not observed (*n* = 2 *Ptf1a*^*CreERTM/+*^ pancreata). These observations indicate that *Ptf1a*^*CreERTM*^*-*mediated recombination is pancreatic acinar cell-specific and occurs only after tamoxifen treatment.

### Mature acinar cells expressing oncogenic alleles of *Kras* and *Trp53* initiate the formation of desmoplastic and metastatic PDAC

**Fig 2:**
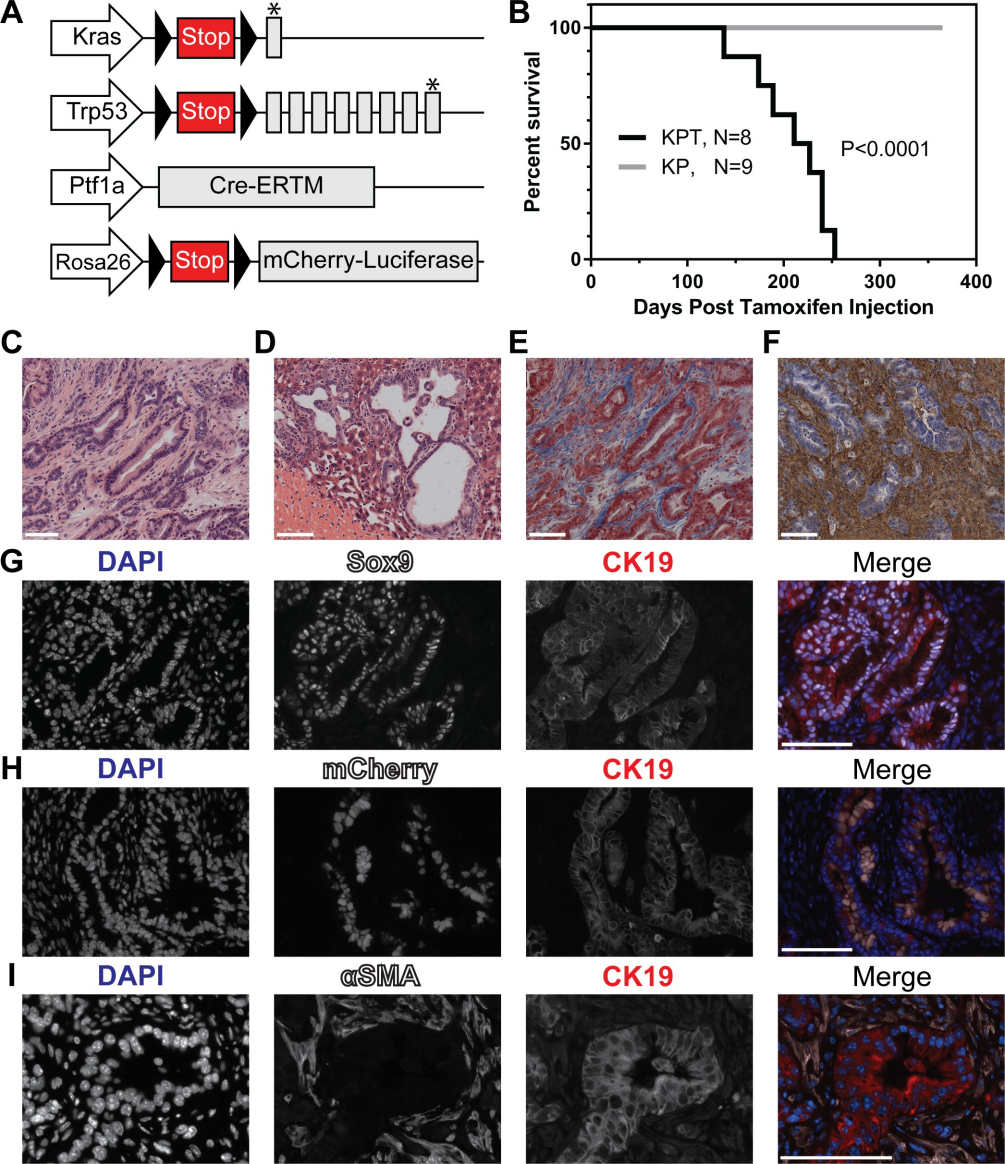
KPT mice develop metastatic and desmoplastic PDAC. **(A)** Graphic representation of alleles altered in KPT mice. Triangles represent LoxP sites. Arrows indicate genetic loci. Grey boxes indicate exons. Asterisks indicate point mutations. **(B)** Kaplan-Meier survival analysis of KPT and KP mice post tamoxifen injection. Median survival for KPT mice is 219 day post tamoxifen injection. *P* < 0.0001 by means of the log-rank test. Representative micrographs depicting H&E stained sections of PDAC **(C)** and liver metastasis **(D)** in KPT mice. **(E)** Representative micrographs of Trichrome stained tissue sections of PDAC in KPT mice (*n* = 5). **(F)** Representative micrographs depicting histochemical visualization of HA in KPT tumors, HA is stained brown, nuclei are counterstained blue (*n* = 5). Magnification of brightfield images is 20x. **(G)** Multiplexed IF staining of KPT tumors for detection of nuclei (DAPI, blue), Sox9 (white) and CK19 (red). *n* = 3 KPT tumors, magnification 40x. **(H)** Multiplexed IF staining of KPT tumors for detection of nuclei (DAPI, blue), mCherry (white) and CK19 (red) *n* = 2 KPT tumors, magnification 40x. **(I)** Multiplexed IF staining of KPT tumors for detection of nuclei (DAPI, blue), αSMA (white) and CK19 (red) *n* = 3 KPT tumors, magnification 63x. All scale bars represent 100 μm.

To determine the capacity of pancreatic acinar cells to initiate the development of PDAC, 4–5-week-old C57BL/6j mice with the genotype *Ptf1a*^*CreERTM/+*^; *Kras*^*LSL-G12D/+*^*; Trp53*^*LSL-R270H/+*^*; Rosa26*^*LSL-mCL/LSL-mCL*^ (KPT; **Fig 2A**) were injected with tamoxifen and monitored for tumor development to a humane study endpoint. KPT mice developed PDAC with a median survival of 219 days post-tamoxifen injection (Total *n* = 8 KPT mice [male: *n =* 3; female: *n =* 5]; **Fig 2B**). Littermate control mice lacking an inducible Cre recombinase (genotype *Ptf1a*^*+/+*^; *Kras*^*LSL-G12D/+*^*; Trp53*^*LSL-R270H/+*^*; Rosa26*^*LSL-mCL/LSL-mCL*^; or KP) were injected with tamoxifen and monitored for tumor development for up to one year. No pancreatic tumors were observed in KP mice (*n* = 9 KP mice). Hematoxylin and eosin (H&E)-stained histological sections of KPT pancreatic tumors show morphological features typical of PDAC with infiltrating, well- to poorly-formed glands displaying marked cytological atypia and high mitotic activity. Invasion was associated with a marked desmoplastic response within the involved pancreatic parenchyma and the involved peripancreatic fat and duodenal mucosa (**Fig 2C**). Macrometastases of PDAC to the liver and lungs were observed in 3/8 and 1/8 KPT mice respectively during necropsy (**Fig 2D**).

PDAC tumors resected from KPT mice were examined for evidence of tumor fibrosis and molecular markers of ductal cell phenotype. The histochemical visualization of hyaluronan (HA) and the Masson’s Trichrome Stain were used to qualitatively measure the deposition of extracellular matrix components in KPT tumors. Trichrome stained sections confirmed abundant collagen deposition within KPT tumors, and the concurrent presence of HA was confirmed by histochemical visualization (**Fig 2E and 2F**). IF staining was used to examine cancer cells in KPT tumors for the expression of molecular markers of ductal cell phenotype. KPT tumors were observed to express Sex-Determining Region Y-Box 9 (Sox9), and CK19; both proteins are well characterized molecular markers of PDAC (**Fig 2G**, *n* = 3 tumors) [29,30]. To determine if cancer cells in KPT tumors were directly descended from mature pancreatic acinar cells, IF staining against the mCL lineage marker was performed on KPT tumors. Multiplexed IF staining of KPT tumors identified mCherry^+^/CK19^+^ cancer cells, while CK19^-^ stromal cells were also mCherry^-^ (**Fig 2H**, *n* = 2 tumors). The presence of activated cancer associated fibroblasts (CAFs) was evaluated via multiplexed IF staining of alpha-smooth muscle actin (αSMA), a marker of activated myofibroblasts. IF staining reveals the presence of activated CAFs infiltrating KPT tumors (**Fig 2I**, *n* = 3 KPT tumors). This staining result indicates that KPT tumor cells express molecular markers of ductal morphology and are directly descended from mCL positive pancreatic acinar cells. Additionally, KPT tumors are infiltrated by activated CAFs, and are characterized by the deposition of the fibrotic extracellular matrix components HA and collagen. In summary, the expression of *Kras*^*G12D*^ and *Trp53*^*R270H*^ by mature pancreatic acinar cells is sufficient to initiate the development of PDAC which recapitulates key attributes of human PDAC including highly differentiated, desmoplastic, and metastatic tumors.

### Oncogene-expressing mature acinar cells undergo ADM prior to PDAC formation

**Fig 3:**
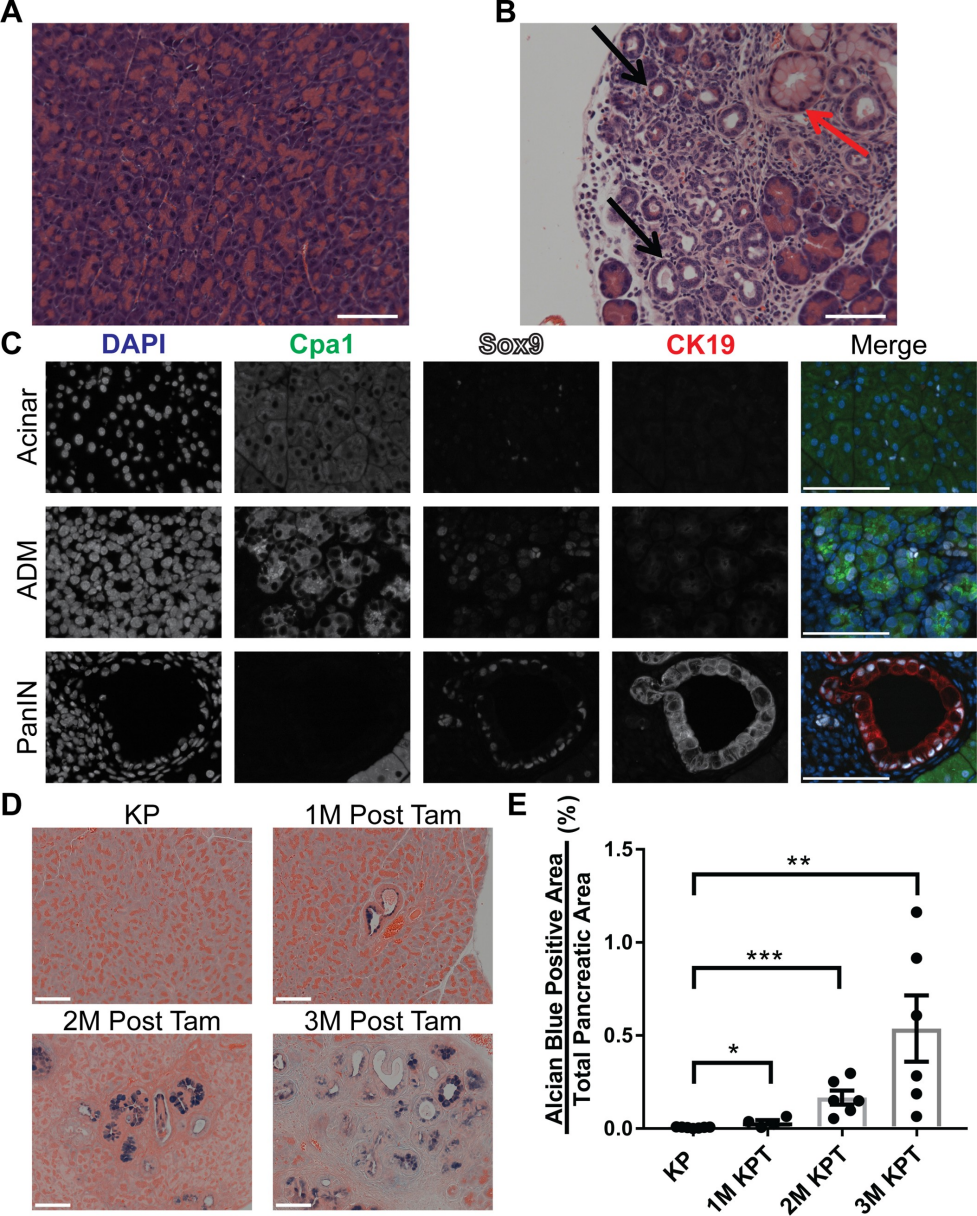
ADM and PanIN lesions precede tumor formation in KPT mice. **(A-B)** Representative micrographs depicting H&E stained sections of the pancreata of KP **(A)** and KPT **(B)** mice 3 months post tamoxifen treatment. Black arrows indicate regions of acinar to ductal metaplasia, red arrow indicates PanIN lesion. (*n* = 6 KPT mice, and *n* = 7 KP mice), magnification 20x. **(C)** Representative multiplexed IF micrographs of KPT pancreata 2 months post tamoxifen treatment, for detection of nuclei (DAPI, blue), Cpa1 (green), Sox9 (white) and CK19 (red). Images depict acinar tissue with normal histology (upper panels), an isolated region of early acinar to ductal metaplasia (middle panels) and a PanIN lesion (lower panels), *n* = 6 KPT mice, magnification is 63X. **(D)** Representative micrographs of alcian blue and eosin (AB/E) stained sections of the pancreata of KPT and KP littermate control mice 1M, 2M, and 3M post tamoxifen injection, magnification is 20X. **(E)** Whole slide quantification of alcian blue positive area relative to eosin positive total pancreatic area, data points represent 1 AB/E stained slide per mouse, *n* = 7 for KP mice, *n* = 4 for 1M KPT mice, *n* = 6 for 2M KPT, *n* = 6 for 3M KPT. Bars depicts mean with error bars representing standard error of the mean (SEM). Significance was determined by student’s *t* test. *, *P* < 0.05; **, *P <* 0.01; ***, *P <* 0.001. All scale bars represent 100 μm.

Previous studies have demonstrated that acinar cell-derived PDAC is preceded by ADM and PanIN lesion formation [10,11]. We sought to examine the progression of PDAC from acinar cell-derived precursor lesions in the KPT model; and to determine if the development of pancreatic cancer precursor lesions precede tumor formation in the pancreata of KPT mice. We examined the pancreata of KPT mice which were euthanized prior to tumor development for evidence of ADM and PDAC precursor lesion formation. KPT and KP mice were euthanized 1, 2 and 3 months post-tamoxifen treatment (1M, 2M, and 3M). Examination of H&E-stained histological slides of the pancreata of control KP mice identified no pathological abnormalities such as regions of ADM or PanIN lesions, defined here as flat to architecturally complex lesions composed of columnar to rounded atypical nuclei associated with mucin production (*n* = 4 1M KP, *n* = 6 2M KP, *n* = 6 3M KP; **Fig 3A**). H&E staining of the pancreata of KPT mice revealed regions of acinar cells undergoing ADM at all time points (**Fig 3B**). Sox9 is normally expressed only by healthy duct cells and centroacinar cells, and the aberrant expression of Sox9 by acinar cells is a requirement for ADM [31,32]. We used multiplexed IF staining to identify acinar cells undergoing ADM, indicated by positivity for both ductal and acinar cell markers. Healthy acinar cells displaying normal morphology were Cpa1^+^/Sox9^-^/CK19^-^ with isolated Sox9^+^ nuclei in the exocrine pancreas putatively indicating centroacinar cells or intercalated ducts (**Fig 3C**, top panels). Regions of ADM (Cpa1^+^/Sox9^+^; **Fig 3C,** middle panels), and PanIN lesions (Cpa1^-^/Sox9^+^/CK19^+^; **Fig 3C**, bottom panels) were observed in the pancreata of KPT mice. These results show that the expression of *Kras*^*G12D*^ and *Trp53*^*R270H*^ is sufficient to induce mature acinar cells to undergo ADM, and demonstrate that the KPT model may be used to study factors driving the formation and progression of these precursor lesions. Moreover, transformed acinar cells present in regions of ADM in the KPT mice are putative tumor-initiating cells and therefore, could be used as a model to study strategies to selectively target this population.

### The abundance of pre-cancerous PanIN lesions in KPT mice increases during the PDAC latency period

We measured the relative frequency of pre-cancerous PanIN lesions in KPT mice prior to tumor formation by histological examination of the pancreata of 1M, 2M and 3M KPT mice. When compared to benign acini, PanIN lesions are characterized by cytoplasmic mucin. The Alcian blue histological stain detects acidic mucins and has been used to accurately measure the frequency of PanIN lesions in similar transgenic mouse models of PDAC [11,31], although acidic mucin production can be present in reactive, non-neoplastic, ductal lesions [33,34]. Alcian blue-positive PanIN lesions were not observed in the pancreata of KP littermate control mice at any time point (**Fig 3D**, top left panel) consequently, all KP littermate cohorts were grouped for statistical analysis. Alcian blue-positive lesions were detected in KPT mice at all time points (**Fig 3D**). Relative quantification of PanIN lesions, determined by measuring the quantity of a given pancreatic section which is Alcian blue-positive divided by total pancreatic area, reveals an increasing quantity of PanIN lesions in KPT mice as time from tamoxifen treatment lengthens (**Fig 3E**). These observations indicate that the relative abundance of PanIN lesions in KPT mice increases longitudinally post tamoxifen treatment, thus accurately recapitulating the stepwise progression of PDAC from PanIN lesions.

### Transcriptional profiling of regions of ADM and PDAC identifies conserved differentially expressed genes and pathways

**Fig 4:**
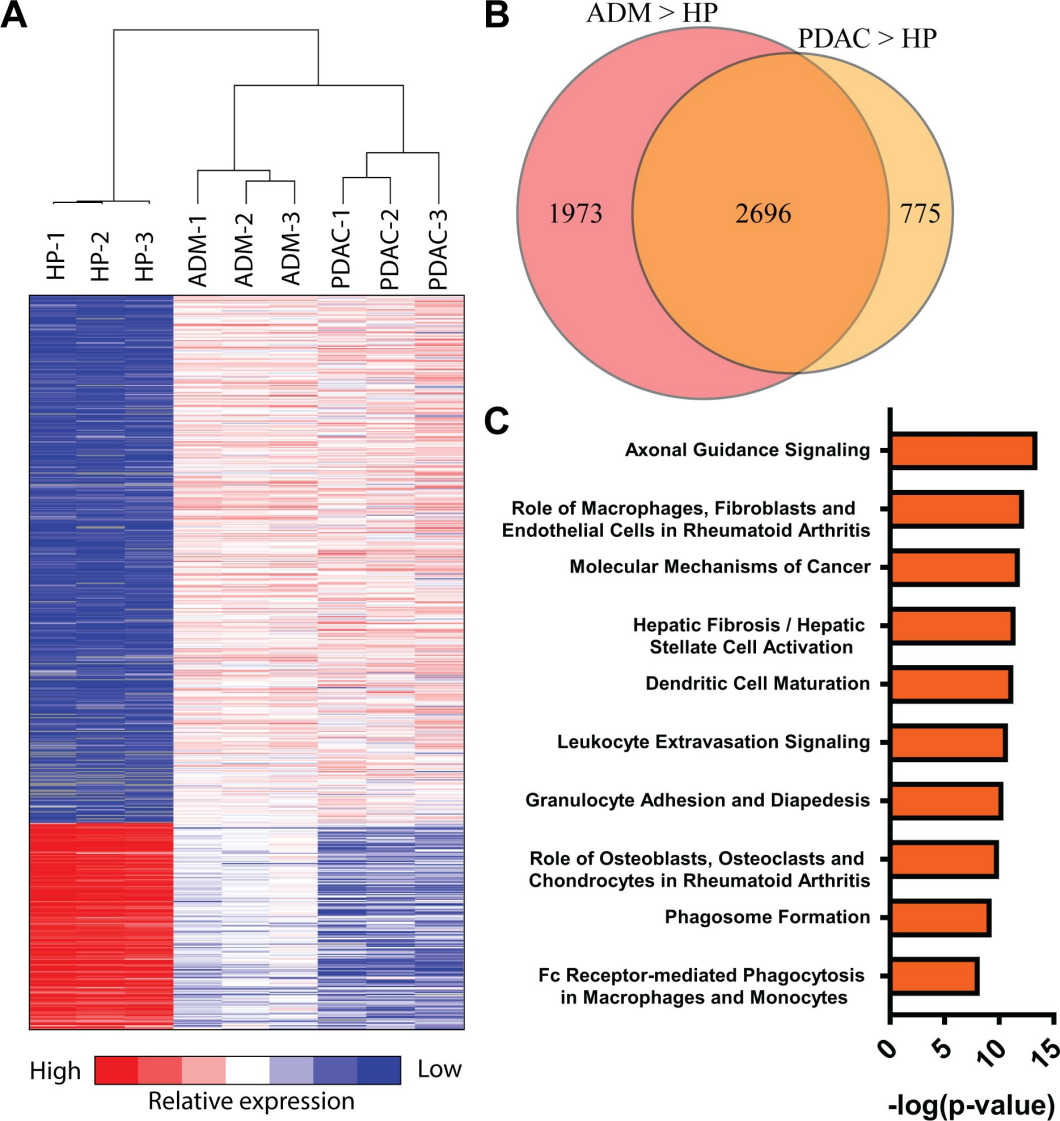
RNA sequencing of microdissected regions of ADM and PDAC identifies differentially expressed genes and pathways. **(A)** Heatmap depicting normalized gene expression of differentially expressed genes in three way comparison between HP, ADM and PDAC samples. **(B)** Venn diagram depicting overlap between differentially expressed genes in HP-ADM and HP-PDAC pair wise comparisons. **(C)** IPA canonical pathway analysis identifies conserved inflammation associated canonical pathways present in ADM and PDAC. Data is represented as negative log (*P* value) of canonical pathway association.

To identify potential biomarkers of early stage oncogene-driven PDAC we generated a comprehensive transcriptome profile of isolated regions of ADM. LCM was used to isolate metaplastic ductal epithelial cells, as well as any mesenchymal or immune cells infiltrating regions of ADM, from the pancreata of 2M KPT mice (ADM, *n* = 3). LCM was used to isolate invasive ductal epithelial cells from pancreatic tumors, which were resected from KPT mice (PDAC, *n* = 3). RNA was extracted from a control population of healthy pancreatic cells from 2M KP mice (HP, *n* = 3). RNA extracted from HP, ADM, and PDAC populations was subjected to RNA-Seq and the results were used to identify differentially expressed genes (DE). Principal component analysis (PCA) of gene expression data revealed similarity in the gene expression profiles between biological replicates within each RNA-Seq sample groups and clearly distinguished sample groups from one another (**S1 Fig**). Differential gene expression analysis comparing the HP and ADM samples identified 1592 upregulated and 503 downregulated genes with expression fold change > 8 and adjusted *P* value < 0.001. Comparing HP and PDAC samples using identical fold change and *P* value thresholds revealed 1217 upregulated and 820 downregulated genes (**Fig 4A**). Validation of RNA-Seq data was conducted by real-time qPCR (**S2 Fig**). IPA software used these lists of differentially expressed genes to predict upstream transcriptional regulators activated or repressed in ADM relative to HP using these lists of DE genes (**S3 Table**).

We identified genes that were up-regulated in both PDAC and ADM samples relative to HP. Differential gene expression analysis identified a total of 5444 genes with > 2 fold greater expression in either ADM or PDAC compared to HP with an adjusted *P* value < 0.01, 2696 genes were identified as differentially expressed in both PDAC and ADM relative to HP (**Fig 4B,** a full list of differentially expressed genes is available in **S4 Table**). These comparisons identified genes overexpressed in both regions of ADM and in PDAC, indicating they are potential biomarkers of PDAC. Conserved canonical signaling pathways activated in both ADM and PDAC samples were identified using IPA software (**Fig 4C**). Canonical pathways identified by IPA include the recruitment and migration of leukocytes and the activation of hepatic stellate cells, which are similar to pancreatic stellate cells (PSC) [35]. These results suggest that the signaling pathways responsible for maintaining the inflammatory and desmoplastic tumor microenvironment present in PDAC may be activated in regions of ADM. Importantly, whole transcriptome analysis by RNA-Seq of PDAC lesions from the KPT model shows high concordance with a recently published data from a human dataset [36], with 5 out of the 10 activated canonical pathways overlapping between the datasets (Table 1). Comparison of KPT tumor RNA with the human dataset shows conserved activation of inflammation and fibrogenesis-related pathways. While these experiments identify genes and pathways which are potential biomarkers of early PDAC oncogenesis, additional experiments are necessary to validate the differential expression of ADM associated genes and demonstrate that these genes influence PDAC initiation.

**Table 1:** Top 10 signaling pathways associated with PDAC in the KPT mice (A) and humans [36] (B), compared to normal murine or human pancreas, respectively. There is a 50% overlap (bold) between the top 10 signaling pathways in the KPT mice and humans, including the top pathway hit.

A) KPT PDAC	-Log (P value)	Ratio	B) Human PDAC	-Log (P value)	Ratio
**1. Granulocyte Adhesion and Diapedesis**	10.517	0.235	**1. Granulocyte Adhesion and Diapedesis**	7.07	0.186
**2. LPS/IL-1 Mediated Inhibition of RXR Function**	7.053	0.185	**2. Inhibition of Matrix Metalloproteases**	5.22	0.308
3. Role of Osteoblasts, Osteoclasts and Chondrocytes in Rheumatoid Arthritis	6.463	0.176	**3. LPS/IL-1 Mediated Inhibition of RXR Function**	4.93	0.151
**4. Hepatic Fibrosis / Hepatic Stellate Cell Activation**	5.804	0.182	4. Antigen Presentation Pathway	4.68	0.297
**5. Agranulocyte Adhesion and Diapedesis**	5.157	0.173	5. Complement System	4.68	0.297
6. Retinol Biosynthesis	5.08	0.31	6. Leukocyte Extravasation Signaling	4.59	0.152
7. Role of Macrophages, Fibroblasts and Endothelial Cells in Rheumatoid Arthritis	4.629	0.144	**7. Hepatic Fibrosis / Hepatic Stellate Cell Activation**	4.41	0.153
**8. Inhibition of Matrix Metalloproteases**	3.973	0.282	8. Cell Cycle Control of Chromosomal Replication	4.36	0.333
9. Axonal Guidance Signaling	3.711	0.124	**9. Agranulocyte Adhesion and Diapedesis**	4.15	0.148
10. Valine Degradation I	3.667	0.389	10. Mitotic Roles of Polo-Like Kinase	3.98	0.212

### The accumulation of HA occurs in conjunction with ADM, and HA deposition precedes collagen deposition in PDAC precursor lesions

**Fig 5:**
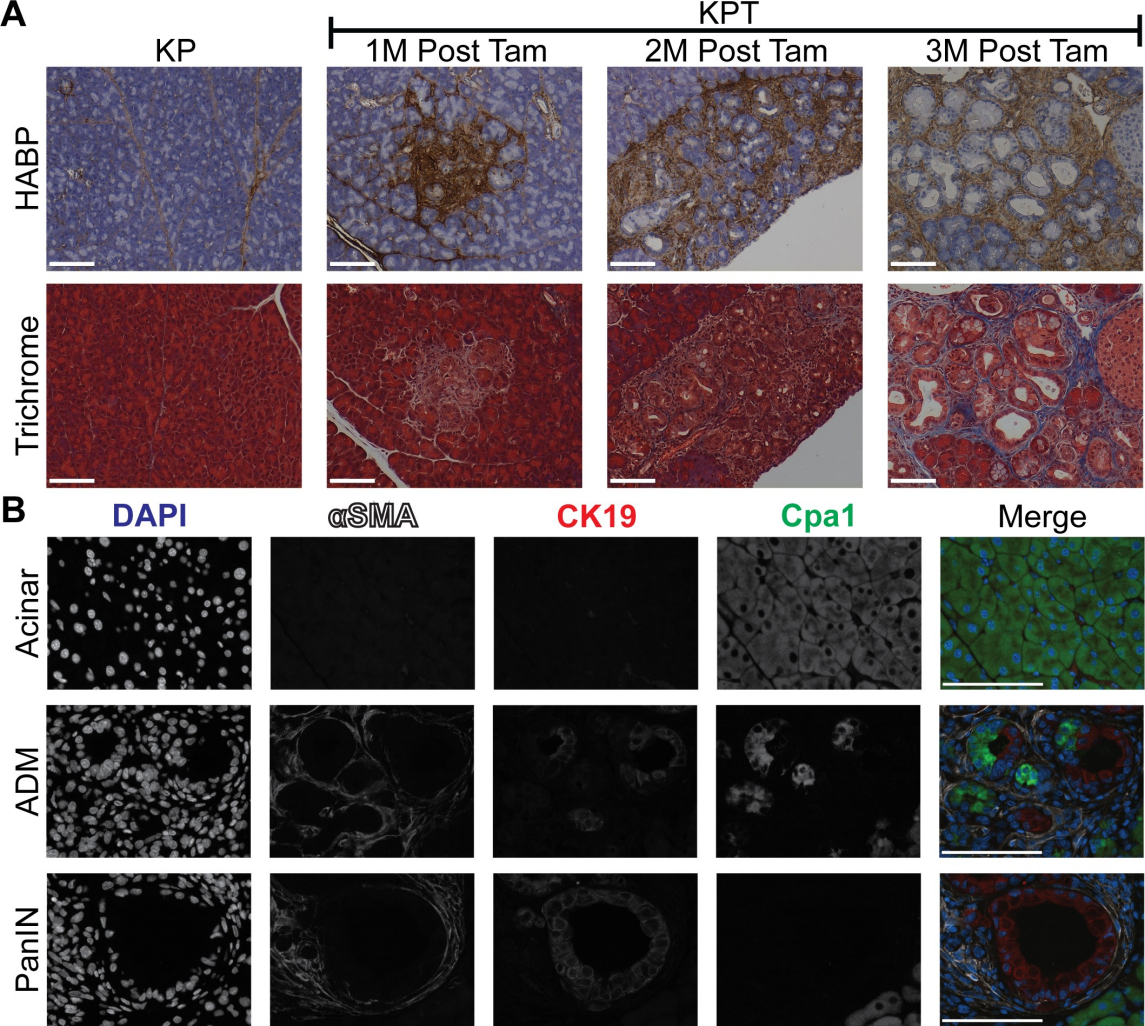
The desmoplastic response occurs in conjunction with ADM and increases with progression to PanIN. **(A)** Histochemical visualization of HA (brown) counterstained with hematoxylin (blue). Representative micrographs depict the pancreata of KPT and KP mice 1 month, 2 months, and 3 months post-tamoxifen injection. Images of KPT pancreata depict dysplastic regions containing ADM and/or PanIN. Masson’s trichrome staining depicting collagen deposition (blue) in serial sections from the same KPT pancreata as shown in **(A)**, *n* = 7 for KP mice, *n* = 4 for 1M KPT mice, *n* = 6 for 2M KPT, *n* = 6 for 3M KPT. Magnification of bright field images is 20X. **(B)** Representative multiplexed IF micrographs for detection of nuclei (DAPI, blue), αSMA (white), CK19 (red), and Cpa1 (green). Rows depict healthy acinar tissue (acinar), a region of ADM and PanIN lesions. Micrographs are representative of the pancreata of KPT mice 2 months post tamoxifen treatment (*n* = 3), magnification is 63X. All scale bars represent 100 μm.We sought to identify the source of HA in regions of ADM, via the detection of activated PSC. Increased activation and proliferation of PSC are directly involved in the development of PDAC associated fibrosis via the deposition of ECM components including HA and collagen in the tumor microenvironment [40]. Multiplexed IF staining against αSMA, CK19 and Cpa1 revealed the presence of activated PSC in proximity to PanIN lesions and infiltrating regions of ADM in KPT mice. Activated αSMA-positive PSCs were not observed in regions of Cpa1^+^/CK19^-^ healthy acinar tissue (**Fig 5B**, top panel). Activated PSC were observed infiltrating regions of ADM containing Cpa1^+^/CK19^+^ cells undergoing ADM (**Fig 5B**, middle panel), and CK19^+^/Cpa1^-^ PanIN lesions were surrounded by activated PSC (**Fig 5B**, bottom panel). These observations indicate that PSC recruitment and/or activation occur during ADM and that neoplasia-associated HA production and PSC activation precede collagen deposition, which is associated with PanIN lesions. To our knowledge, the role of desmoplasia in general, or HA deposition specifically, in the initiation of PDAC has not been investigated in detail, due to the paucity of animal models. While PSC activation in regions of ADM is associated with HA deposition, additional experiments are necessary to definitively identify the specific cellular origin of HA in regions of ADM. These results establish the KPT model as an ideal model to study the role of the desmoplastic response in PDAC development from a defined population of adult cells.

*Kras*^*G12D*^ driven ADM is potentiated by inflammatory, tissue-remodeling macrophages, but the specific changes in ECM composition responsible for increasing the frequency of ADM lesions are unknown [37–39]. To identify changes in ECM components associated with ADM, we compared the expression of ECM constituents in healthy pancreas and microdissected regions of ADM. Differential gene expression analysis comparing HP and ADM sample groups revealed an increase in the expression of hyaluronan synthase genes (Has1-3), and of the hyaluronan receptors (CD44, Stab2, and Hmmr) in regions of ADM (**Table 2**). The expression of collagens 1, 3–6, 8–12, 13–16, 18, & 23–27 was up-regulated in regions of ADM relative to HP, while the expression of collagen 7 was down regulated in regions of ADM relative to HP (**S5 Table**). We sought to validate these observations via the histochemical visualization of HA and collagen in the pancreata of 1M, 2M and 3M KPT mice. Intralobular deposition of HA was observed in regions of ADM and in proximity to PanIN lesions as early as one month post tamoxifen (**Fig 5A**, top panels). In both KPT and KP mice, HA staining was not observed in histologically normal pancreatic acinar lobules, but physiological HA staining was observed in parenchymal tissue and along the borders of acinar lobules. Trichrome staining indicates limited accumulation of collagen in the proximity of ADM or PanIN lesions in the pancreata of 1M and 2M KPT mice. However, PanIN lesions in the pancreata of 3M KPT mice were positive for collagen deposition (**Fig 5A**, bottom panels). We found no evidence of the deposition of supraphysiological collagen in the acinar lobules in KP mice at any time point. These results indicate that the deposition of HA occurs in conjunction with ADM and that the accumulation of HA precedes collagen accumulation during the development of PDAC.

**Table 2:** The expression of hyaluronan related genes is increased in regions of ADM. Differential gene expression analysis comparing ADM and HP sample sets identifies HA related genes. Significance is defined as log_2_ (fold change) > 1 and an adjusted *P* value < 0.001, all else is defined as not significant (NS).

Gene Category	Gene Name	Gene Symbol	log_2_ (Fold Change)	Adjusted *P* value	HP VS ADM (Up/NS)
Hyaluronan Synthase	Hyaluronan Synthase 1	Has1	2.43	1.09E-02	NS
	Hyaluronan Synthase 2	Has2	4.10	2.52E-09	Up
	Hyaluronan Synthase 3	Has3	1.87	4.01E-05	Up
Hyaluronidase	Hyaluronoglucosaminidase 1	Hyal1	0.70	4.52E-03	NS
	Hyaluronoglucosaminidase 2	Hyal2	1.31	3.01E-08	Up
	Hyaluronoglucosaminidase 3	Hyal3	-1.00	1.52E-01	NS
Hyaluronan Receptor	CD44 Molecule (Indian Blood Group)	Cd44	5.35	1.83E-120	Up
	Hyaluronan Mediated Motility Receptor	Hmmr	1.28	2.14E-03	Up
	Stabilin 2 (Hyaluronan Receptor For Endocytosis)	Stab2	3.89	8.80E-04	Up

## Discussion

In this study, we describe an autochthonous, genetically engineered, mouse model of adult acinar cell-derived pancreatic cancer. We provide evidence that the KPT model of pancreatic cancer recapitulates all stages of the progression model of pancreatic cancer in which an adult cell acquires genetic mutations, forms pancreatic cancer precursor lesions and ultimately develops into metastatic PDAC [41,42]. The KPT model of PDAC is then utilized to characterize the transcriptional changes and ECM remodeling which is associated with ADM and PDAC progression following the expression of oncogenic alleles of *Kras* and *Trp53* by mature pancreatic acinar cells.

The KPT mouse model of PDAC utilizes the activity of a well-characterized inducible Cre recombinase to restrict the expression of oncogenic alleles of *Kras* and *Trp53* to the adult pancreatic acinar cell compartment [22]. Acinar cell specific expression of oncogenes is a significant temporospatial departure from the “Gold standard” preclinical mouse model of PDAC known as the KPC model; in which Cre recombinase knocked into the *Pdx1* locus activates the expression of oncogenic alleles of *Kras* and *Trp53* in the fetal pancreas [21,43]. Moreover, compared to the KPC model where tumors may originate from multiple cellular lineages, PDAC cells in the KPT model are specifically descended from transformed acinar cells. Because KPT mice develop PDAC from a specific cellular population, the KPT mouse model could be used as a tool for the examination of factors which influence PDAC initiation from mature acinar cells.

The study of PDAC precursor lesion formation is relevant to the study of the human disease because pancreatic precursor lesions may influence metastasis. In transgenic mice, pancreatic epithelial cells originating in preinvasive pancreatic cancer precursor lesions can survive in blood circulation and colonize the liver, potentiating later metastasis [44,45]. This population of circulating precursor cells is evolutionarily and phenotypically distinct from metastatic pancreatic cancer cells. The KPT model is suitable for the study of the impact of PDAC precursor lesions on subsequent PDAC metastasis. The evaluation of putative pancreatic cancer precursor lesions in PDAC is complicated by the fact that the precise cellular origin of PDAC in humans is not yet known.

Two separate research groups have recently investigated the cell of origin for PDAC by deleting *Trp53* and activating the expression of Kras^G12D^ separately in the adult pancreatic acinar or ductal lineages [10,11]. In both of these lineage tracing investigations, pancreatic acinar cells were capable of initiating PDAC development and this manuscript presents an independent validation of the tumor initiating capacity of mature acinar cells. The KPT model can be distinguished from similar acinar cell derived models of PDAC because of the luciferase reporter expressed by PDAC cells. This bioluminescent reporter permits the longitudinal monitoring of precursor lesion formation and tumor progression in living mice. The KPT model of adult acinar cell-derived carcinogenesis constitutes a platform for pre-clinical studies examining the progression of acinar cell-derived PDAC from the earliest precursor lesions.

Previously generated transcriptional profiles of ADM have used total pancreatic RNA isolated from transgenic mice to represent ADM [46–48]. These transcriptome profiles utilized chemical agents to induce acute pancreatitis in oncogene-driven, genetically engineered mouse models of PDAC. Consequently, these RNA-seq experiments fail to distinguish between gene expression signatures produced by cells undergoing transient, pancreatitis-induced ADM and the transcripts produced by cells undergoing irreversible, oncogene-induced ADM. The transcriptome profile of ADM presented in this manuscript is a more accurate representation of early pancreatic carcinogenesis than previous profiles specifically because it examines a highly enriched population of cells undergoing ADM. Additionally the use of LCM to isolate regions of ADM (instead of a method which isolates a relatively pure population of cells such as fluorescence activated cell sorting) ensures that the transcriptional profile of ADM will identify genes produced by any non-acinar cells which specifically infiltrate regions of ADM. This transcriptional profile of regions of ADM, which specifically includes ADM infiltrating cells, may lead to the identification of additional paracrine signaling pathways required for PDAC oncogenesis. However RNA expression profiles from heterogeneous, LCM-isolated samples are incapable of identifying the specific cell type in which a given gene of interest is differentially expressed. Consequently further experiments are necessary to identify the cell type in which ADM associated genes are differentially expressed. The KPT model of PDAC is well suited for use in experiments which examine the factors required for ADM since specific cellular populations may be depleted, or signaling pathways blocked, prior to the induction of oncogene expression.

Kras^G12D^-driven ADM is potentiated by inflammatory, ECM-remodeling macrophages, which are recruited to regions of ADM in an intercellular adhesion molecule-1 (ICAM1)-dependent mechanism [39]. However the precise ECM factors which enhance ADM are unknown. The transcriptome profile of regions of ADM generated in this manuscript identifies ECM factors which may potentiate ADM, but further rigorous experiments are required to conclusively demonstrate a causative relationship between the expression of specific ECM components and ADM.

Oncogene-driven ADM is the earliest characterized physiological change preceding PDAC development. Consequently, the observation that ADM and desmoplasia are concurrent events challenges canonical models of tumor initiation in which the desmoplastic reaction occurs as a response to invasion of tissue by tumor cells. These results clearly demonstrate that acinar cell-derived precancerous lesions are preceded by desmoplasia, but further studies are necessary to determine if precancerous lesions which are descended from ductal cells display a similar desmoplastic profile. Comparative analysis of these disparate PDAC precursor lesions may elucidate the signaling pathways that regulate oncogene induced activation of PSC and the accompanying desmoplastic response.

The function and cellular source of fibrotic HA deposition in the initiation and progression of ADM lesions has not yet been investigated in detail. However, oncogene-driven ADM resembles acute inflammatory pancreatic injury and is potentiated by acute pancreatitis [31,38,49,50]. HA content more than doubled 24 hours after the induction of pancreatitis in a rodent model of acute pancreatitis. [51]. HA production in both acute pancreatitis and ADM may be involved in a fibrotic response which restricts the deregulated release and diffusion of cytotoxic pancreatic digestive enzymes. Alternatively, the HA receptor CD44 is a marker of cancer stem cells and tumor initiating cells in PDAC. CD44 signaling is implicated in the dissemination of cancer cells during metastasis, which may occur during ADM or pancreatic cancer precursor lesion formation [52–54]. Further experiments are required to determine if HA signaling through CD44 or other pathways is required for ADM and PanIN formation. These independent observations suggest a relationship between HA deposition and the recruitment of immune cell populations to regions of ADM. Regions of ADM are characterized by the recruitment of inflammatory immune cells including macrophages, and HA is involved in the recruitment and activation of macrophages [37,55].

Macrophages could also be a source of HA in regions of ADM. Pancreatic tissue-resident, tumor-associated macrophages express hyaluronan synthase 2 and 3 and display a pro-fibrotic phenotype in transgenic mouse models of PDAC [56]. Experiments in which the expression of hyaluronan synthase is inhibited in specific cellular lineages, such as PSCs, macrophages, or acinar cells, could identify the source of HA in regions of ADM. The inducible nature of the KPT animal model makes it appropriate for investigating the source and role of HA in ADM. The influence of HA deposition on the progression of oncogene-driven ADM could be evaluated in KPT animals by treating these mice with a hyaluronidase enzyme such as PEGPH20 following tamoxifen treatment [16,20].

In summary, we demonstrate that the KPT model of pancreatic cancer is a useful tool for studying the initiation and progression of acinar cell-derived PDAC. The KPT model of pancreatic cancer is characterized by the stepwise initiation of PDAC from healthy, mature acinar cells to ADM lesions, to PanINs and ultimately to invasive, metastatic PDAC [41]. We use the KPT mouse model of PDAC progression to characterize the initiation of desmoplasia during ADM; specifically, to demonstrate that HA is deposited in the earliest pancreatic cancer precursor lesions. These experiments demonstrate the utility of the KPT model of PDAC for future studies examining: (1) the role of desmoplasia in the development of pancreatic cancer precursor lesions; (2) how the oncogenic transformation of acinar cells alters the basal ECM to facilitate malignant transformation; (3) the identification of biomarkers of pancreatic cancer progression; and (4) the preclinical evaluation of strategies to disrupt the tumor-initiating capacity of acinar cells to mitigate metastasis.

## Supporting information

S1 Fig Principal component analysis reveals overall similarity between sample subsets. (PDF)

S2 Fig Real time qPCR of selected genes validate the results of RNA-Seq analyzes. (PDF)

S1 Table RNA and RNA-Seq quality control. (PDF)

S2 Table Kras and Trp53 mutational analyzes to confirm that transcriptomics were performed on true lesions. (PDF)

S3 Table IPA Prediction of Upstream transcription regulators: HP to ADM comparison. (XLSX)

S4 Table Complete list of differentially expressed genes identified by HP-ADM/PDAC comparison. (XLSX)

S5 Table The expression of collagens is increased in regions of ADM. (XLSX)
