# Supplementary material for: Desmoplasia and oncogene driven acinar-to-ductal metaplasia are concurrent events during acinar cell-derived pancreatic cancer initiation in young adult mice
Source: PLoS One. 2019 Sep 6;14(9):e0221810. doi: 10.1371/journal.pone.0221810 (PMC6731019; doi:10.1371/journal.pone.0221810)
Supplement: S1 Fig — (PDF) [file pone.0221810.s001.pdf]

S1

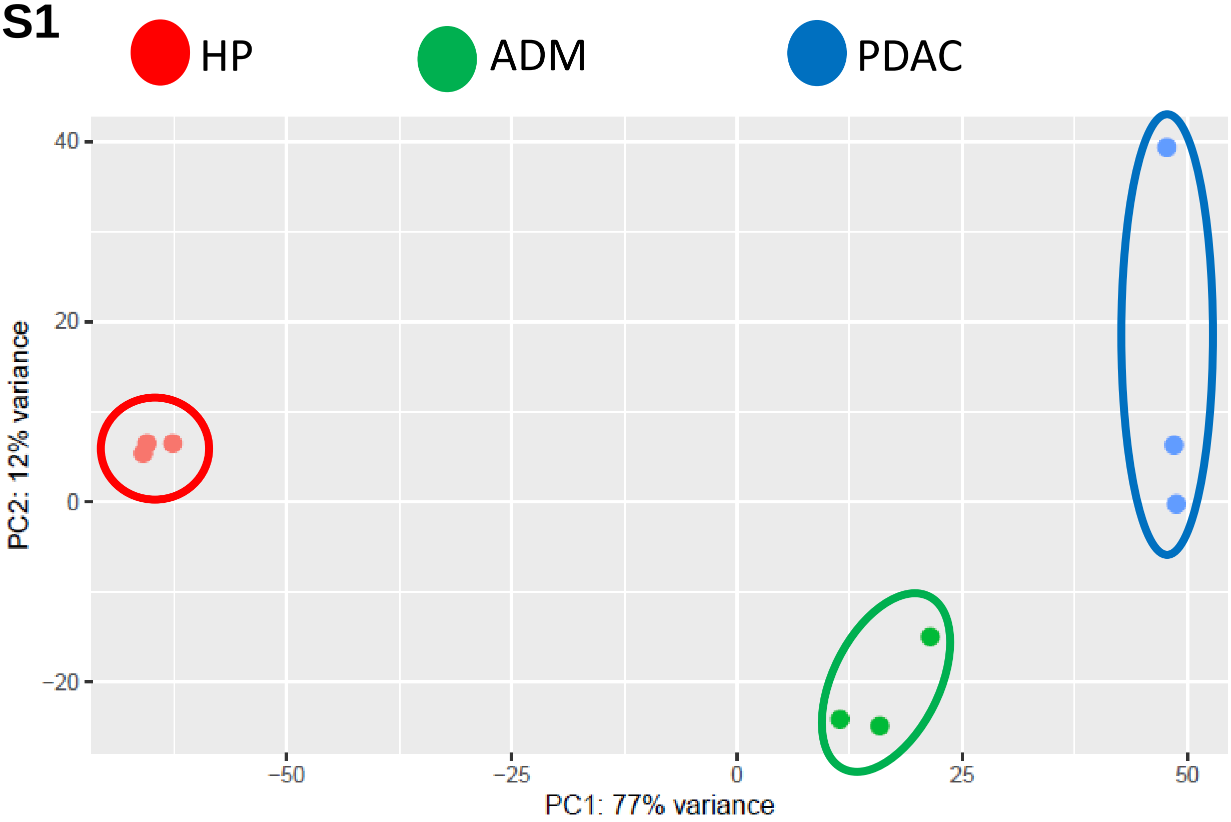

Principal component analysis reveals overall similarity between sample subsets
Principal Component Analysis (PCA) performed using DESeq2 log-normalized RNA-seq data for all samples to evaluate the overall effect of experimental covariates. The biological triplicates for each sample set are shown in the two-dimensional plane spanned by their first two principal components (PC1 and PC2). Each axis represents the proportion of variation explained by each of the principal components. The biological triplicates of each sample subset (HP, ADM and PDAC) are represented as dots and are grouped together in the PCA plot, sample groups are clearly separated.
