# Supplementary material for: Desmoplasia and oncogene driven acinar-to-ductal metaplasia are concurrent events during acinar cell-derived pancreatic cancer initiation in young adult mice
Source: PLoS One. 2019 Sep 6;14(9):e0221810. doi: 10.1371/journal.pone.0221810 (PMC6731019; doi:10.1371/journal.pone.0221810)
Supplement: S2 Fig — (PDF) [file pone.0221810.s002.pdf]

S2

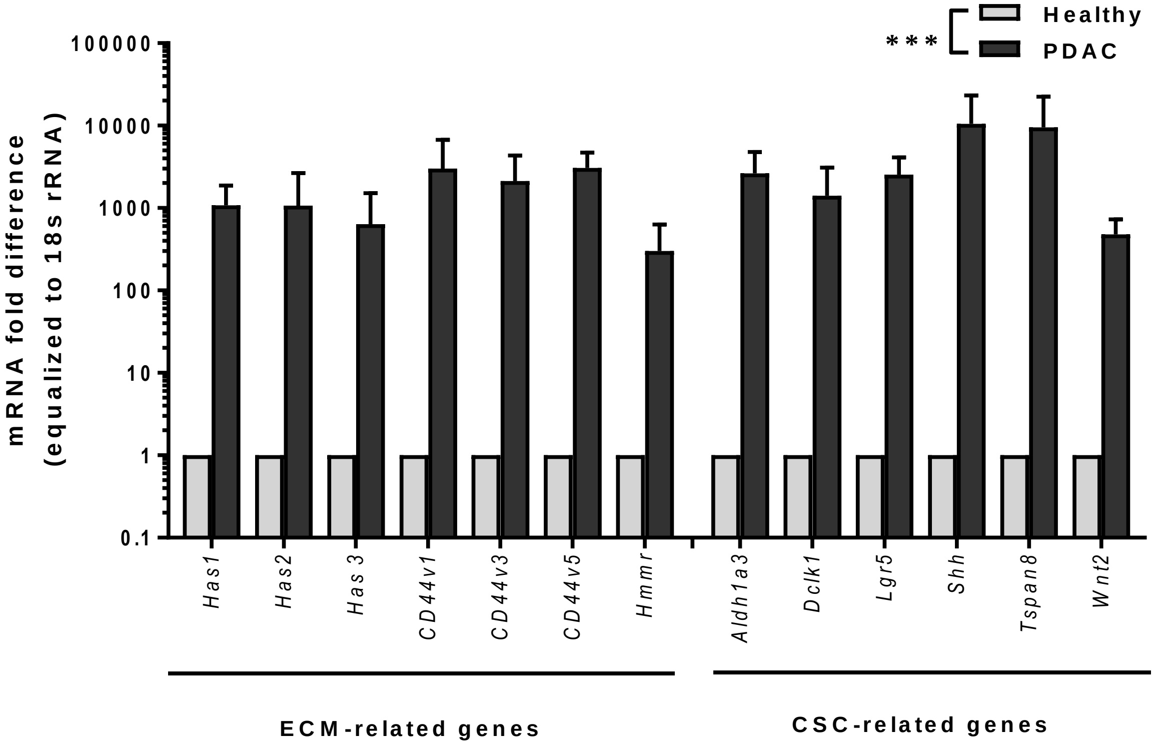

qRT-PCR of selected genes validate the results of RNA-seq analysis. To confirm the expression profile of genes in RNA-Seq results, 13 genes with known involvement in clinical PDAC progression were selected. Extra-cellular matrix (ECM) and Cancer stem cell-related (CSC) genes were up-regulated in PDAC as compared to healthy pancreas as per the RNA-seq analysis. Methodology: pancreata from KPT mice **1.** Untreated with TAM (normal whole pancreas control; N=3); and **2.** At 5 months post-TAM (frank PDAC, N=3) were harvested, minced into fragments of approximately 2mm^3 using sterile scalps and stored in RNAlater stabilization buffer (QIAGEN) at -80°C until isolation. RNA was isolated using RNeasy extraction kit (QIAGEN). One microgram of RNA was converted to cDNA using the RevertAid First Strand cDNA Synthesis kit from Life Technologies using random hexamer primers. All reactions were carried out according to manufacturer's protocols. Gene expression analyses were performed in triplicate using the QuantStudio 3 Real-Time PCR system (Life Technologies) with SYBR Green (Applied Biosystems, Life Technologies). Target genes were amplified using the following conditions: 50°C for 2 minutes, 95°C for 10 seconds, followed by 40 cycles of 95°C for 15 seconds; 60°C for 1 minute. 18S rRNA was used as a house keeping gene to normalize for cDNA input. Results from N=3 mice/group were combined and expressed as fold change compared to whole normal pancreas control. Statistical analysis of relative gene expression was performed using the comparative ΔΔCT method and significance determined by using ANOVA followed with Bonferroni correction on Excel. Primers were purchased from Integrated DNA Technologies (IDT). Primer sequences used are listed in the table below:

Target gene	Primer sequence 5’-3’
Aldh1a3	F: GGGTCACACTGGAGCTAGGA
	R: CTGGCCTCTTCTTGGCGAA
Dclk1	F: TCCACCGGAATTGAACTCGG
	R: GGGAGCGAACAGTCTCAGA
Lgr5	F: CCTACTCGAAGACTTACCCAGT
	R: GCATTGGGGTGAATGATAGCA
Shh	F: AAAGCTGACCCCTTTAGCCTA
	R: TTCGGAGTTTCTTGTGATCTTCC
Tspan8	F:TCTGGGTATGTGGTACACTGAT
	R: AGGGGTTCGTGCTAGAGTCTC
Wnt2	F: CTCGGTGGAATCTGGCTCTG
	R: CACATTGTCACACATCACCCT
Has1	F: GGCGAGCACTCACGATCATC
	R: AGGAGTCCATAGCGATCTGAAG
Has2	F: TGTGAGAGGTTTCTATGTGTCCT
	R: ACCGTACAGTCCAAATGAGAAGT
Has3	F: GTGGGCACCAGTCTGTTTG
	R: CCACTGAACGCGACCTCTG
CD44v1	F: CACCATTGCCTCAACTGTGC
	R: TTGTGGGCTCCTGAGTCTGA
CD44v3	F: TCGATTTGAATGTAACCTGCCG
	R: CAGTCCGGGAGATACTGTAGC
CD44v5	F: TCTGCCATCTAGCACTAAGAGC
	R: GTCTGGGTATTGAAAGGTGTAGC
Hmmr	F: CCTTGCTTGCTTCGGCTAAAA
	R: CTGCTGCATTGAGCTTTGCT
