# Supplementary material for: Desmoplasia and oncogene driven acinar-to-ductal metaplasia are concurrent events during acinar cell-derived pancreatic cancer initiation in young adult mice
Source: PLoS One. 2019 Sep 6;14(9):e0221810. doi: 10.1371/journal.pone.0221810 (PMC6731019; doi:10.1371/journal.pone.0221810)
Supplement: S1 Table — (PDF) [file pone.0221810.s003.pdf]

S1 table. RNA-Seq quality control data analyzes

Sample I.D.	RIN	Total_Reads	Overall_Mapping%	Multi_Align%	Exonic_Read_Counts	Exonic%
1. Normal pancreas	7	54713579	90.1	33.3	29656140	60.1
2. Normal pancreas	7.7	51163117	91.5	34	27630333	59
3. Normal pancreas	6.9	53700967	86.5	34.2	26697504	57.4
4. ADM	6.2	51971440	90.5	9.2	25701289	54.6
5. ADM	5.8	51266351	89.9	9.7	23903780	51.8
6. ADM	7.4	55271381	89.7	8.1	27482854	55.4
7. PDAC	8.7	51520029	91.6	7.3	28838890	61.1
8. PDAC	7.5	54021906	92.8	6.8	28946686	57.7
9. PDAC	9.1	55337629	91.5	7.5	31315456	61.8

RIN (RNA integrity number) was used as a metric to assess RNA quality. RIN>5 was considerable suitable for the analysis, considering the challenges involving extraction of high-quality RNA by LCM from pancreatic tissues. Alignment statistics shows good alignment rate (~90%>) for all samples, with satisfactory percentage of mapped reads at exons (~50-60%).
