# Supplementary material for: Desmoplasia and oncogene driven acinar-to-ductal metaplasia are concurrent events during acinar cell-derived pancreatic cancer initiation in young adult mice
Source: PLoS One. 2019 Sep 6;14(9):e0221810. doi: 10.1371/journal.pone.0221810 (PMC6731019; doi:10.1371/journal.pone.0221810)
Supplement: S2 Table — (PDF) [file pone.0221810.s004.pdf]

S2 Table. *Kras* and *Trp53* Mutational analyzes to confirm that transcriptomics were performed on true lesions

Trp53:c.G809A:p.R270H	Chromosome 11<br>Pos 69589608	REF<br>G	ALT<br>A	Alt. Allele Freq
1. Normal pancreas		2	0	0.00%
2. Normal pancreas		0	0	NA
3. Normal pancreas		4	0	0.00%
4. ADM		39	8	17.02%
5. ADM		20	13	39.39%
6. ADM		56	14	20.00%
7. PDAC		4	1	20.00%
8. PDAC		10	35	77.78%
9. PDAC		20	96	90.57%

Kras:c.G35A:p.G12D	Chromosome 6<br>Pos 145246771	REF<br>C	ALT<br>T	Alt. Allele Freq
1. Normal pancreas		0	0	NA
2. Normal pancreas		0	0	NA
3. Normal pancreas		0	0	NA
4. ADM		3	2	40.00%
5. ADM		1	13	92.86%
6. ADM		4	9	69.23%
7. PDAC		4	3	42.86%
8. PDAC		4	1	20.00%
9. PDAC		7	14	66.67%

To confirm whether LCM microdissected ADM and PDAC lesions expresses mutant *Trp53R270* and *KrasG12D*, and therefore are true lesions, abundance of cDNA fragments which contain the oncogenic point mutations relative to the abundance of cDNA which encodes for wildtype *Trp53* and *Kras* sequences in ADM and PDAC samples were measured and compared to normal pancreas. As expected, Trp53R270H and KrasG12D mutations (top and bottom table, respectively) are present in the ADM/PDAC subsets, but were not detected in the healthy pancreas samples. Methodology: Samtools was used to compile a table of nucleotide base coverage for R270H in *Trp53* and G12D in *Kras* for each sample. An in-house program written in C++ was used to collect the depth of each allele and calculate the alternative allele frequency.
